# Relationship between Glycosylated Hemoglobin, Serum Nitric Oxide and Mean Arterial Blood Pressure

**Published:** 2014-12

**Authors:** M Manju, Sasmita Mishra, B. D. Toora, Vijayakumar, R Vinod

**Affiliations:** 1 Department of Biochemistry, Aarupadai Veedu Medical College, Pondicherry, India;; 2 Department of Microbiology, SriVenkateswara Medical College Pondicherry, India

**Keywords:** Nitric oxide, glycosylated hemoglobin, arterial blood pressure

## Abstract

**Background:**

Hypertension is about twice as frequent in individuals with diabetes as in those without diabetes. Formation of glycosylated conjugates like HbA1c is implicated to have many effects on the vascular endothelium which leads to the development of hypertension in diabetes. Nitric oxide (NO)-dependent vasodilatation has been shown to be an important factor in the maintenance and regulation of peripheral vascular tone. Studies correlating these parameters give conflicting results. Hence the present study was designed to correlate HbA1c, Serum NO & mean arterial blood pressure.

**Aims:**

To study the relationship between glycosylated hemoglobin, serum nitric oxide & mean arterial blood pressure.

**Settings and Design:**

It is a case control study with 28 type 2 diabetic hypertensives, 32 type 2 diabetic normotensives and 51 controls (non diabetic normotensives).

**Materials & Methods:**

The study subjects included 28 type 2 diabetic hypertensives, 32 type 2 diabetic normotensives and 51 controls (non diabetic normotensives) [ADA 2010 and JNC7]. FBS, PPBS, PCV, Hb, HbA1c & serum NO estimation and BP recording was done in all the study subjects. Normalised mean arterial blood pressure (MAPn) and calculated glycosylated hemoglobin (cHbA1c) were calculated from mean arterial BP (MAP) and HbA1c respectively.

**Statistical Analysis:**

was done using R commander software. The difference in the distribution of cHbA1c, MAPn & NO levels between all 3 groups was measured using ANOVA and Kruskal-Wallis test. Correlation between the parameters was measured by Correlation coefficient and logistic regression (Spearman linear regression) analysis (univariate and multivariate).

**Results:**

There was a significant difference in the distribution of cHbA1c, MAPn & NO levels (p<0.001) between all 3 groups, whether measured by ANOVA or Kruskal-Wallis test. On univariate analysis, there was a positive correlation between cHbA1c & MAPn (ρ= +0.26), a negative correlation between NO & MAPn (ρ = -0.54) and cHbA1c & NO (ρ= -0.66) .On multivariate analysis, not only NO, but contrary to univariate analysis, cHbA1c also showed a negative association with MAPn.

**Conclusion:**

As the severity of diabetes increases, there is increase in BP, which is mainly due to the marked decrease in NO level which masks the negative correlation between HbA1c on MAPn.

## INTRODUCTION

Hypertension is about twice as frequent in individuals with diabetes as compared to those without diabetes (1). One of the major complications of diabetes is vascular disease, (micro and macro vascular) which is considered to be one of the leading causes of death in diabetes patients. The vascular complications include stroke, coronary artery disease, peripheral vascular disease, retinopathy, nephropathy and possibly neuropathy. Many observational studies have shown that people with both diabetes and hypertension have approximately twice the risk of cardiovascular disorders as non-diabetic people with hypertension. Further, it has been investigated that hypertensive diabetic patients are at enhanced risk for retinopathy and neuropathy. Multiple factors are probably involved in sustaining elevated BP in DM.

The raised arterial pressure in type 2 diabetes is attributed to the endothelial dysfunction and pressor effects of hyperinsulinemia and hyperglycemia. Formation of glycosylated conjugates like HbA1c is implicated to have many effects on the vascular endothelium which may lead to the development of hypertension in diabetes. The normal endothelium plays an important role in maintaining vessel wall homeostasis, synthesizing biologically active substances that modulate vascular tone (2). Important among these vasoactive substances is endothelium-derived relaxing factor (EDRF), identified as nitric oxide, or a related nitroso compound that liberates nitric oxide (3). NO is a paracrine mediator acting as a potent vasodilator in various vascular beds. NO is synthesized as a by product of conversion of its physiological precursor L-arginine to L-citrulline. This reaction is catalyzed by a family of enzymes known as NO synthases (NOS) (4). NO-dependent vasodilatation has been shown to be one of the important factors in the maintenance and regulation of peripheral vascular tone. Although the available data are conflicting, a number of studies have demonstrated impaired vascular response to endothelial dependent vasodilators or reactive hyperemia in diabetic patients suggesting impaired production or enhanced degradation of nitric oxide or depressed smooth muscle responses to nitric oxide. The mechanisms underlying this abnormality in vascular function are not well established (5). With this background, this study was designed to study the relationship between glycosylated hemoglobin, serum nitric oxide and mean arterial blood pressure.

## MATERIALS & METHODS

It was a case control study conducted in the Dept. of Biochemistry, A. V. Medical College, after obtaining the approval from scientific research committee of our college. The study subjects included age and sex matched 28 type 2 diabetic hypertensives, 32 type 2 diabetic normotensives and 51 controls (non diabetic normotensives).Diabetes and hypertension was diagnosed based on ADA 2010 (6) and JNC7 (7) criteria.. All the study subjects were from the patients who attended the General Medicine OPD of our college. Exclusion criteria included Type I diabetes mellitus, gestational DM, alcoholics (>30 gm/week), chronic inflammatory diseases, IHD, stroke, neoplasms, renal diseases, women on oral contraceptive pills or steroids and whose heart rate >100. The aim and procedures were explained to patients and/or attendant and informed consent was obtained.

Blood pressure was recorded using standard mercury sphygmomanometer twice with a gap of 2 hours in rt. arm sitting position (after 10 min rest). BP was recorded again after one week. Fasting venous blood (5 ml) sample were taken from all the subjects included in the study. Hypertension was defined as the blood pressure ≥140/90 mm Hg. Parameters measured in blood include fasting blood sugar (glucose oxidase method)-IL Aries fully autoanalyser, 2 hours postprandial sugar, hemoglobin, hematocrit (Hct), serum Nitric oxide (Griess reaction method) (8), HbA1c (ion exchange resin column-Human).

Mean arterial pressure was calculated using the formula MAP=Diastolic BP + 1/3 (systolic BP-Diastolic BP). MAP was normalized* vs. hematocrit as follows. MAPn = MAPi × (Hct_ave_/Hct_i_). Normalization was done to exclude the effects of blood viscosity on MAP variability due to distribution of Hct on the population. % of HbA1c was converted to an absolute concentration as follows. CHbA1ci = % HbA1c × 13(Hct_i_/Hct_ave_) g/dL.

## RESULTS

Statistical analysis of the results was done by R commander software. Results are presented as mean ± 2SD. Statistical significance and difference from control & test values were evaluated by ANOVA & Kruskal Wallis test. Correlation coefficient and logistic regression analysis (univariate and multivariate) were used to describe the relationship between HbA1c, NO and MABP. Scattergram was plotted by taking dependent variables on y-axis and independent variable (NO) on x-axis.

Table 1 shows the comparison of various parameters in different study groups. The diastolic BP, MAP, MAPn, FBS, PPBS & MBG were raised significantly in diabetic hypertensives and diabetic normotensives as compared to the normal subjects. There was a significant raise in MAPn & MBG in diabetic hypertensives as compared to diabetic normotensives also. The HbA1C (%) & CHbA1C (g/dl) were raised significantly in diabetic hypertensives as compared to diabetic normotensives and normal subjects. NO values were significantly decreased in diabetic hypertensives as compared to both normal subjects as well as diabetic normotensives.

**Figure 1:**
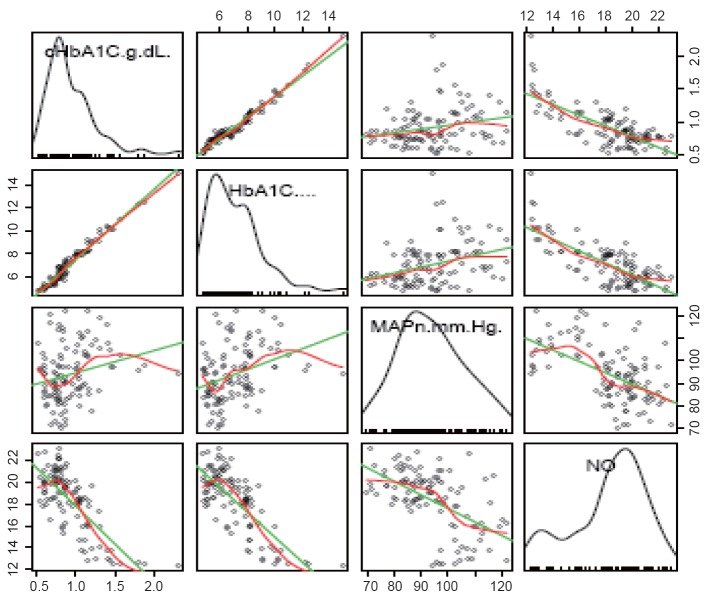
Total scatter plot showing the correlation between HbA1c, cHbA1c, MAPn & NO.

**Figure 2:**
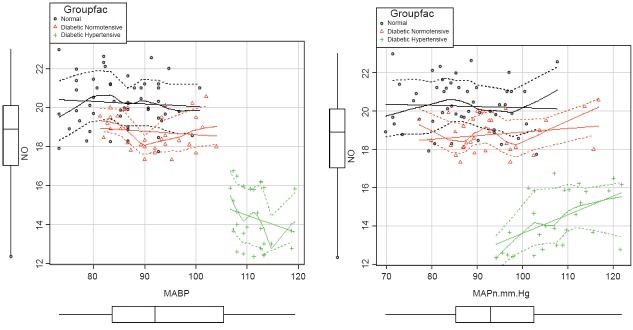
Scatter plot showing the correlation between NO and MABP & MAPn.

There was a strong negative correlation between NO and MAPn as well as between CHbA1C and NO levels (Fig. 1 and Fig. 2). That means, as NO level increases, the MAPn decreases. Similarly, as CHbA1C level increases, NO level was found to decrease. There was a positive correlation between CHbA1C and MAPn. Multivariate analysis was also done (Table 2).

**Table 1:** Comparison of parameters in different study groups

Parameters	Control (n=51)	Diabetic normotensives (n=32)	Diabetic hypertensives (n=28)

**Age (years)**	49.21 ± 22.6	51.75 ± 16.8	51.64 ± 18.2
**SystolicBP(mm Hg)**	117.13 ± 20	122.94 ± 19	146.35 ± 14* †
**DiastolicBP(mmHg)**	69.37 ± 12	75.5 ± 13*	94.28 ± 12* †
**MAP (mm Hg)**	85.46 ± 14	91.3 ± 13*	111 ± 7* †
**MAPn(mm Hg)**	87 ± 18	93 ± 19*	108 ± 15* †
**FBS (mg/dL)**	84.2 ± 28.5	127.81 ± 62*	155.18 ± 90*
**PPBS(mg/dL)**	115.31 ± 29.3	220.62 ± 93*	262.93 ± 120*
**MBG(mg/dL)**	108.23 ± 33	170.53 ± 43*	217.35 ± 126.8* †
**HbA1c (%)**	5.57 ± 1.06	7.53 ± 1.38*	9.07 ± 3.8* †
**CHbA1c (g/dL)**	0.71 ± 0.19	0.96 ± 0.28*	1.24 ± 0.68* †
**NO((μmol/L)**	20.25 ± 2.6	18.77 ± 1.8*	14.34 ± 2.89* †
**PCV**	38.51 ± 5.2	38.31 ± 4.7	40.46 ± 5.2

* *p*<0.001 as compared to control;

† *p*<0.001 as compared to diabetic normotensives.

**Table 2:** Univariate analysis by spearman linear regression

Parameters	Correlation coefficient (ρ)

cHbA1c and MAPn	+ 0.26
NO and MAPn	- 0.54
cHbA1c and NO	- 0.66

## DISCUSSION AND CONCLUSION

Our study results conclude that there is a significant correlation between NO and BP even when adjusted for HbA1c levels. There is a significant difference between NO levels in controls and diabetic normotensives. This implies that endothelial dysfunction (in the form of decreased NO) starts even in diabetic normotensives. Diabetic normotensives are at a higher risk to develop essential hypertension in the future. So careful monitoring of BP for diabetic normotensives is very important. Logically, an intervention to increase NO like regular exercise or supplementation by arginine can be recommended in these people. There is now substantial evidence that endothelium-dependent vasodilation is abnormal in animal models of diabetes mellitus (9).

Impaired NO production can cause hypertension by raising renal and systemic vascular resistances and by promoting sodium and water retention. In addition, NO deficiency can promote glomerulosclerosis and arteriosclerosis by facilitating cell migration and proliferation within the glomeruli and blood vessel walls (10). Moreover, loss of the inhibitory effect of NO on platelet adhesion can facilitate the formation of microthrombi and the release of thrombogenic, proinflammatory, and mitogenic platelet factors. Thus, NO deficiency can impair renal and vascular function and structure.

The significant difference in cHbA1c between diabetic normotensives and diabetic hypertensives can be attributed to an effect of glycosylated hemoglobin (besides the vascular effects) on development of hypertension in diabetes. As the severity of diabetes increases, there is increase in BP, which is mainly due to the marked decrease in NO level which masks the negative correlation between cHbA1c on MAPn as evidenced by multivariate analysis (Fig. 2).

The results of our study support the idea that diabetes mellitus affects the vascular endothelium & the associated vascular tone by affecting NO levels. Vascular endothelium has now received the status of an “organ”. In DM, there is dysfunction, dysregulation & failure of vascular endothelium. NO is a potent vasodilator, synthesised when physiological L-arginine is converted to L-citrulline by NO synthetase enzyme. The decrease in NO level in diabetic hypertensives may be due to 1) inactivation of NO synthetase. (Reduced kidney function as part of aging or due to kidney dysfunction, which is accelerated by diabetes, may prevent the elimination of the major NOS inhibitor, ADMA, thereby limiting the production of NO (11); 2) inactivation of NO by reactive oxygen species produced by HbA1c which damage the vascular endothelium (12); 3) Glycosylated hemoglobin binds NO in the form of nitroso thiols very tightly so that any NO that is formed cannot be easily released from RBC to help maintain blood flow through smooth muscle cell relaxation; 4) Direct effect of very high levels of glucose on vascular endothelium leading to significant blunting of NO response; 5) Insulin resistance of type2 DM i.e. insensitivity of endothelium to insulin action causes increased peripheral resistance and hence rise in BP (13). [Insulin itself is an endothelium dependent vasodilator.]

## FUTURE PROSPECTS OF THE STUDY

A follow up (cohort) study of the diabetic normotensives every 6 months with NO levels and see how many develop hypertension and how it is related to the duration and severity of diabetes mellitus. Effect of exercise & L-arginine supplementation on NO levels can also be done.
